# National strategy for integrating palliative care into standard cardiac care for people living with heart failure: a position statement from the joint working group of the Swiss Societies of Cardiology and Palliative Care

**DOI:** 10.3389/fcvm.2025.1548595

**Published:** 2025-03-28

**Authors:** Piotr Z. Sobanski, Maria Luisa De Perna, Sandra Eckstein, Tanja Fusi-Schmidhauser, Jan Gaertner, Valentina Gonzalez-Jaramillo, Lisa Hentsch, Caroline Hertler, Roger Hullin, Lukas Hunziker, Philip Larkin, Jean-Baptiste Mercoli, Philippe Meyer, Giorgio Moschovitis, Matthias Paul, Otmar Pfister

**Affiliations:** ^1^ Palliative Care Station und Kompetenzzentrum, Innere Medizin, Spital Schwyz, Schwyz, Switzerland; ^2^ 1st Department of Cardiology, Medical University of Gdansk, Gdańsk, Poland; ^3^ Ente Ospedaliero Cantonle (EOC), Servizio di Cardiologia, Sede Ospedale Regionale di Lugano, Istituto Cardiocentro Ticino, Lugano, Switzerland; ^4^ Division of Palliative Care, Department Theragnostic, University Hospital Basel, Basel, Switzerland; ^5^ Palliative and Supportive Care Clinic, Ente Ospedaliero Cantonale, Bellinzona, Switzerland; ^6^ Palliative and Supportive Care Clinic, Ente Ospedaliero Cantonale, Lugano, Switzerland; ^7^ Department of Rehabilitation and Geriatrics, University of Geneva, Geneva, Switzerland; ^8^ Palliative Care Center, Bethesda Spital AG, University of Basel, Basel, Switzerland; ^9^ Department of Clinical Research, University of Basel, Basel, Switzerland; ^10^ University Center for Palliative Care, Inselspital, Bern University Hospital, Bern, Switzerland; ^11^ Division of Palliative Care, Department of Rehabilitation and Geriatrics, Geneva University Hospital, Geneva; ^12^ Kompetenzzentrum Palliative Care, Klinik für Radioonkologie, Universitätsspital Zürich, Zürich, Switzerland; ^13^ Service de Cardiologie, Centre Hospitalier Universitaire Vaudois (CHUV), Lausanne, Switzerland; ^14^ Department of Cardiology, Bern University Hospital, University of Bern, Bern, Switzerland; ^15^ Chaire Kristian Gerhard Jebsen de Soins Palliatifs Infirmiers, Universite de Lausanne, Centre Hospitalier Universitaire Vaudois (CHUV), Lausanne, Vaud, Switzerland; ^16^ Palliative Care, Service of Oncology, Gruppo Ospedaliero Moncucco, Lugano, Switzerland; ^17^ Division of Cardiology, Geneva University Hospitals, Geneva, Switzerland; ^18^ Ente Ospedaliero Cantonle (EOC), Servizio di Cardiologia, Sede Ospedale Regionale di Lugano, Istituto Cardiocentro Ticino, Lugan, Switzerland; ^19^ Herzzentrum, Luzerner Kantonsspital, Luzern, Switzerland; ^20^ Department of Cardiology, University Hospital Basel, University of Basel, Basel, Switzerland

**Keywords:** palliative care, heart failure, needs assessment, quality of life, national strategy

## Abstract

**Introduction:**

Heart failure (HF) causes high symptom burden and shortens life expectancy. Implementation of Palliative Care (PC) concurrently with cardiologic guidelines-directed medical therapy (GDMT) improves quality-of-life (QoL) more than disease-oriented management alone but is underused. To facilitate provision of PC for people living with HF, the Swiss Society of Cardiology (SSC) and the Swiss Society for Palliative Care (palliative.ch) have created joint working-group.

**Methods:**

Dyads representing cardiology and PC from Swiss HF centres have been identified. Through online voting, workshops and Delphi process priority topics for incorporation of PC into standard care for people with HF have been identified.

**Results:**

18 experts, from 8 Swiss HF-centres identified main topics relevant for implementation of PC in usual HF care: timely recognition of unaddressed health-related needs of affected people and their relatives (using validated assessment tools ID-PALL or NAT-PD:CH at least as the triggers evolve), identifying key palliative interventions for care of people living with HF, identifying strategies to facilitate cooperation between specialist PC and cardiology, defining research agenda to investigate efficacy of PC interventions, quality of care criteria, and outcomes of PC provision in Switzerland.

**Discussion:**

Improvement of QoL of people with HF and their relatives could be greater if PC would be integrated in usual care timely. Frequent needs assessment, using validated tools helps to recognize people having unaddressed needs, and helps to replace the outdated, based on risk of dying, involvement of PC. Dialogue between both disciplines is crucial to provide care prolonging life of best quality during the whole journey living with disease.

## 1 Introduction

Palliative care (PC) is a concept of care that focuses on improving the quality of life (QoL) of people living with serious, chronic, potentially life-limiting diseases and their relatives by implementing symptomatic interventions. It is usually applied in addition to the disease-specific, guidelines-directed medical therapy (GDMT) provided by other medical specialties. Both palliative and disease-specific approaches aim to improve QoL, but in different ways. A palliative approach is oriented toward the relief of suffering by means of the alleviation of symptoms and problems (e.g., decreasing the sensation of breathlessness and the suffering it causes), while disease-oriented management improves disease-related pathophysiology (by decreasing the stimuli evoking breathlessness, such as congestion or low cardiac output in the case of heart failure, HF). Applying PC and disease-specific interventions concurrently should be understood as setting a meta-goal for comprehensive care, ensuring a life of the best possible length and quality, from the beginning to the very end of living with a serious disease, using all available approaches.

## 2 Methods and consensus definitions

The Swiss Societies of Cardiology (SSC) and Palliative Care (palliative.ch) have created a joint working group to facilitate interdisciplinary exchange, aimed at facilitating the provision of PC for those living with HF, in a uniform way across all regions of the country. The working group stressed the significance of distinguishing between two types of PC when design care: specialist PC (for those with complex and/or persistent problems, provided by or with the engagement of PC specialists/teams) and primary (general) PC (sufficient for the majority, provided by the usual care team which has basic PC training and specialist PC back-up). Once unaddressed needs have been recognized (which can emerge at any time, and do not correspond with either planned management, prognosis or disease progression), they should be evaluated in accordance with existing local structures and standards of care by the team deemed responsible. In most cases, the usual care team (cardiology, internal medicine, geriatrics, general practice) remains in charge of providing comprehensive care, including the elements of generalist PC. PC specialists/teams should be involved as needed, in the form of consultation, engagement with the usual care team, taking the lead in care only occasionally, in complex situations. Regardless of who is providing the PC, needs should be reassessed regularly to ensure they have been addressed appropriately, and in the case of insufficient efficacy, the intensity of collaboration between teams may need to be increased. Adequate multidisciplinary support (which may include medical management, psychosocial service, chaplaincy, and support by allied therapies such as physio- or ergo-therapy) can be more effective than generalist PC in alleviating the burden experienced by patients and their relatives/caregivers. A multidisciplinary PC team provides additional support to the usual care team by facilitating in-depth communication, preparedness for shared decision making, or assisting in advance care planning (ACP). Such collaborative support increases the quality of care, reduces the workload for the general practitioner (GP)/usual care team, and potentially prevents care fragmentation. Moreover, it increases satisfaction with care, and the concordance between care desired and received, especially during the end-of-life (EoL). Patient-reported outcomes and financial issues related to providing PC require further investigation to verify the clinical efficacy and cost-effectiveness of the proposed approach (1–3).

## 3 Results

In the course of a kick-off meeting held during the National SSC Congress in Basel in June 2023, the working group identified the following four most relevant topics for establishing the provision of palliative elements in the standard care for people living with HF:

I. The timely recognition of the unaddressed health-related needs of people living with HF and their relatives (identification of needs assessment tools and triggers to apply them),
II. Identifying key palliative interventions for the care of people living with HF,
III. Identifying strategies to facilitate cooperation between specialist PC and cardiology,
IV. Defining research agenda to investigate the efficacy of PC interventions, quality of care criteria, and outcomes of PC provision in Switzerland.

### 3.1 The timely recognition of unaddressed health-related needs of people living with HF and their relatives (needs assessment tools and triggers to apply them)

#### 3.1.1 Needs assessment tools

Early integration of PC into disease management strategies alleviates disease-related suffering and improves QoL. For this reason, the World Health Organization (WHO) recommends the timely inclusion of PC in the standard care pathways for people living with any potentially life-limiting disease, including cardiovascular diseases, with HF as a key example (1, 4–6). Despite this, to date PC integration into care for those living with non-oncological disease has been scarce. The major barriers prohibiting early integration are misconceptions of PC as being appropriate for those who are imminently dying and/or are suffering from oncological diseases only (7). However, almost every person living with HF cannot be cured, despite receiving optimal GDMT, and can be confronted with limitations to the length and quality of life. Thus, they should be considered as candidates for receiving PC. Providing optimal, comprehensive care for all people living with HF without delay requires the timely and reliable recognition of unmet needs and the initiation of appropriate interventions. The working group has confirmed such a strategy as appropriate and applicable for people living with HF in Switzerland and has identified the crucial meaning of the timely recognition of unaddressed needs using validated tools. To choose the most suitable needs assessment tools for people living with HF, an additional workshop, held during the National Palliative Care Congress in Biel in November 2023, and two rounds of using the Delphi process were needed. 21 health care professionals in the first Delphi round and 17 in the second (both nurses and physicians), working in different settings of cardiology and PC in all regions of Switzerland made their recommendations. From the set of needs assessment tools (8–17) (Supplementary Figure S1), the experts proposed two as being the most appropriate for gauging whether those living with HF could have unmet needs and potentially require PC interventions: the IDentification of patients in need of PALLiative care (ID-PALL) and the Needs Assessment Tool: Progressive Disease—Heart Failure (NAT:PD-HF). Both tools are simple, based on clinical evaluations (neither requires special tests), and can be easily applied by health care professionals (mostly nurses or physicians) without special training in PC. ID-PALL (Supplementary Figure S2) has been designed to identify those living with non-malignant or malignant (i.e., oncological) disease who might require PC support in a different acute medical care setting (except in intensive care units and emergency departments) and is available in English, French, and Italian (18–20). NAT:PD-HF (Supplementary Figure S3) has been specifically designed to target needs in HF in any settings of care. It is available in English and has been validated in German (17, 21). The working group has undertaken efforts to make both tools available in all of the national languages of Switzerland. ID-PALL guides evaluation regarding which form of PC should be provided—general or specialized, whereas NAT:PD-HF includes a question on who is needed to address the recognized needs.

ID-PALL uses the surprise question (SQ) as one of the main criteria—a question which healthcare professionals should ask themselves: “would you be surprised if the patient died in the next ….” between 3 and 12 moths has been proposed, with one year being most commonly used (22, 23). NAT:PD-HF contains four sections (patient wellbeing, caregiver wellbeing, their ability to provide care, and issues suggesting a high probability of a need for PC referral) with prompts aiding the evaluation of unmet needs. The tool does not contain any questions on disease progression, prognosis, or risk of death, as studies during the validation process of this tool indicated that needs do not correlate with disease progression.

The SQ, sometimes proposed as a single indicator for the probability of the need for PC, should be seen as more difficult to apply than it seems, as it is neither reliably sensitive (11.6%–95.6%) nor specific (13.8%–98.2%). It carries very high inter-observer differences, making it unreliable and rather difficult to use, especially for health care professionals not trained in providing EoL care and to assess people living with a non-oncological disease, having unpredictable trajectory (22, 24). Additionally, the SQ recognizes the risk of dying and not the need for care, and the risk of death-based initiation of PC is considered outdated and no longer recommended by PC societies, the WHO, and the latest American Heart Association Guidelines on Heart Failure (1, 2). Some experts propose using the SQ as the absolute minimum when more sensitive and validated tools have not been applied, as an indicator who might be in need. Choosing SQ strengthen the stigmatization of PC as care for those at risk of dying. The authors of this statement promote the incorporation of PC at an early stage of living with HF and to perceive the SQ as a way that could help identify those who might require PC urgently due to short life expectancy.

#### 3.1.2 Triggers for performing an assessment

After identifying the needs assessment tools, the second most important step in setting up PC provision for people living with HF is to define when to apply them. The working group proposes that needs should be assessed (using the suggested, validated tools: ID-PALL or NAT:PD-HF) every time there is a significant change in:

- The patient's disease trajectory: e.g., repeated, unscheduled HF-related hospitalizations, emergency room or outpatient visits, considering candidacy for high-risk interventions—mechanical circulatory support, MCS, implantation or heart transplantation, considering management influencing trajectory of living—implantation or exchanging of implantable cardioverter defibrillator (ICD), diagnosing HF for the first time, or its end-stage;
- General health condition: e.g., diagnosis of new, potentially serious concomitant diseases);
- Situation of the next family or the caregiver: e.g., serious disease or death of a spouse.

Additionally, needs assessment should eventually become a routine element of yearly HF follow-up, and more often in the case of those with an unstable condition (Table 1).

**Table 1:** Examples of situations that should trigger needs assessment in people living with.

Patient-triggered	Disease-triggered	Caregiver-triggered
Multidimensional symptoms (including psychosocial/emotional, or spiritual)	First diagnosis or advanced stage of HF	Distress
Performance status/ADL—milestones (loss of recreation, home environment, help with self-care)	Progression/re-adjustment of treatment goals	Informational needs
QoL related	Frequent unscheduled HF-related hospitalizations, emergency room or outpatient visits	Significant change in family/relatives influencing their capability to provide care
Decision support/ACP	Planned relevant intervention/high-risk procedure	
	Yearly HF follow-up	
	A new significant comorbidity	

ADL, activities of daily living; ACP, advance care planning; QoL, quality of life.

The person who performs the given assessment should be determined individually in each centre, according to the local functioning standards and capacities. Basic screening can be initiated by e.g., a (HF) nurse, in both in- or out-patient settings. Studies have shown that after obtaining the required competence in using the proposed tools, performing needs assessment does not prolong the time of a consultation or an outpatient visit and provides additional insights into patient's needs (25–27).

The pattern of assessing needs in people living with HF and assignment of responsibility for PC provision, is depicted in Supplementary Figure S4.

### 3.2 Key palliative interventions for the care of people living with HF

The palliative interventions provided, usually concurrently with optimal cardiac management and care, can be divided into those dedicated to: (1) people living with HF, (2) their relatives and informal caregivers, and/or (3) health care professionals (Table 2). The most relevant interventions dedicated to patients include the following:

• In-depth communication,
• Provision of support in decision-making,
• Provision of symptom management,
• Provision of nonmedical support.

**Table 2:** Palliative interventions relevant to caring for people living with heart failure, their relatives, and healthcare professionals.

Palliative interventions (delivered as generalist or specialist PC)		
Care focused on addressing the needs of patients	Care focused on addressing the needs of relatives	Care focused on addressing the needs of healthcare professionals
First contact—informing about the scope of care and support	**Support in providing care** (taking over some elements of care)	**Networking**
Support in decision-making: - Communication on future health, - **Support in defining personal values and goals,** **ACP process**, - Clarifying alternative (less invasive) treatment options, - Clarifying of support in case of rejecting interventions, - Communication on adjusting of implantable device activities (modification of ICD activity, withdrawal of MCS)	Education - On the fundamentals of the disease to promote understanding how symptoms evolve and how can be alleviated at home - On principles of providing care, - On disease decline	Close cooperation (delivering parallel care): - Assistance in shared decision-making - Support in the process of ACP
Medical interventions: - Symptom management - Limiting of therapies (adjustment of treatment after redefining of goals) - Care for the dying - Palliative sedation - Assistance during MCS withdrawal		Education on: - Principles of primary PC, - Recognition of unaddressed needs, - Criteria governing when specialized PC should be involved
Non-medical support: - Psychosocial support, - Spiritual care, - Dignity therapy.	Psychosocial support	
Delivering hospice care		

Bold: most used interventions. ACP, advance care planning; ICD, implantable cardioverter defibrillator; MCS, mechanical circulatory support.

The interventions dedicated to the family and other informal caregivers include support in caring for patients and ensuring the wellbeing of caregivers themselves (Table 2).

### 3.3 Strategies facilitating cooperation between PC and cardiology

Increasing awareness of the impact of PC on the QoL and the benefits of integrating PC early in the HF management by emphasizing that PC complements rather than replaces diesease-modifying therapies among healthcare providers, patients, families, informal caregivers and the general population.

Initiatives:

a. Organising public health campaigns supported by organisations like the Swiss Heart Foundation, to inform affected people and the general population about the benefits of PC.
b. Training for healthcare professionals (eg., cardiologists, internists, GPs) on the basics of PC and on timely recognition of unaddressed needs.

Empowering GPs as primary care providers, who can apply elements of PC, and coordinate collaboration with specialists, including PC.

Initiatives:

a. Training GPs on the identification of unaddressed PC needs, providing primary PC interventions, and recognising triggers for contacting specialized PC.
b. Facilitation a collaborative relationship among GPs and all specialists through regular communication and active involvement in care planning.

Overcoming main barriers to PC integration in HF management which consists of misconeptions about PC and limited access to specialised PC services.

Initiatives:

a. Educating patients and healthcare professionals on modern PC concepts.
b. Adjusting the usual HF-related communication focused on prolonging life and implementing GDMT by incorporating anticipatory communication on diesease progression.
c. Ensuring easy access to PC services for people with HF by referring them to larger centres, utilising teleconsultations, and increasing the availability of PC providers used in caring for individuals with living HF, if such services are lacking in the usual care centres.

Cross-training for interdisciplinary collaboration between cardiology and PC to enhance mutual understanding, cooperation and skills sharing.

Initiatives:

a. Regular case discussions and joint consultations.
b. Developing collaborative care plans clearly defining roles and responsibilities among providers.
c. Ensuring care decisions are guided by patients` preferences and values, with regular reassessments to adapt if they are changing.

Facilitating interdisciplinary collaboration.

Initiatives:

a. Establishing multidisciplinary teams, including PC specialists, cardiologists, GPs, nurses, social workers, psychologists, and other specialists.
b. Streamlining communication pathways and holding regular meetings for information exchange.
c. Prioritizing a person-centred approach by involving patients and families in care discussions to respect their values, preferences are goals.

### 3.4 The research agenda to investigate the efficacy of PC interventions, quality of care criteria and outcomes of PC provision

Research on the management of most relevant (dyspnoea, pain), refractory symptoms (i.e., persisting with inacceptable intensity or duration for the given patient, despite optimal, adequate and GDMT for this person) in patients with HF:

a. The efficacy and safety of non-pharmacological interventions (such as cognitive behavioural training, hypnosis, virtual reality, and physiotherapy),
b. The efficacy and safety of medical management (non-opioids and different opioids).

Comparison of organizational efficacy, quality and cost-efficiency between generalist PC delivered by a cardiology team (usually a cardiology nurse trained in PC with a specialized PC as a back-up in case of the persistence of unaddressed needs), vs. the usual care (PC delivered on demand).

a. Assessment of the utilization of medical resources (GP visits, emergency room visits, and hospital admissions, or intensive care unit admissions, use of invasive care at the EoL), frequency of shared decision-making and advance care planning discussions, concordance between received and preferred care.
b. Assessment of the cost-effectiveness of primary PC with specialist PC back-up vs. usual care (PC delivered on demand).
c. Defining referral criteria/needs assessment tools which are the most appropriate for providing optimal PC. Of particular relevance for Switzerland is the translation of those tools into all of the national languages in order to unify standards throughout the country.

Comparison of clinical efficacy of different forms of PC (primary with specialist back-up vs. standard care: optimal cardiologic care, PC delivered on demand) on the suffering of people living with HF, the burden of their informal caregivers, and satisfaction from care.

a. Review of the literature to compare the satisfaction of patients and informal caregivers with care in different care models.
b. Setting-up an interventional trial to test whether the results from the literature review can be implemented in the clinical reality in Switzerland, including a comparison of the perspective of the individual patient and the care providing team.

## 4 Conclusions

HF is a common and serious condition characterized by progressive decline, high symptom burden, and shortened life expectancy, even with GDMT. HF is thus a disease warranting access to PC. Many affected people could benefit from receiving PC with the improvement of QoL, symptom burden, and support in the psychosocial, existential and spiritual dimensions; however, the need for PC involvement remains underrecognized (28). The Swiss Societies of Cardiology (SSC) and for Palliative Care (palliative.ch) working group has selected tools which should be used to identify unmet needs, proposed triggers when those tools should be applied, and summarized the PC interventions most relevant in care for people living with HF. In-depth communication on living with disease, which prepares affected people for more active participation in medical decision-making (e.g., shared decision making) and the promotion of anticipatory care planning (e.g., ACP) is increasingly important. This improves the concordance between care wished and received, especially at the EoL, and in this way secures greater satisfaction with and from care.

This document encourages collaboration between cardiologists and PC professionals by identifying existing barriers and proposing strategies to overcome them. Further steps in the development of palliative cardiology, such as examining the efficacy of the approach and setting a future research agenda have also been proposed.

## Data Availability

Published with endorsement of the Swiss Society of Cardiology and the Swiss Society for Palliative Care.

## Data availability statement

The original contributions presented in the study are included in the article/Supplementary Material, further inquiries can be directed to the corresponding author.
