# Supplementary material for: National strategy for integrating palliative care into standard cardiac care for people living with heart failure: a position statement from the joint working group of the Swiss Societies of Cardiology and Palliative Care
Source: Front Cardiovasc Med. 2025 Mar 28;12:1548595. doi: 10.3389/fcvm.2025.1548595 (PMC11985515; doi:10.3389/fcvm.2025.1548595)
Supplement: Supplementary file 1 [file Datasheet1.pdf]

Supplementary files

Suppl. Figure 1. Available tools to assess the need for PC provision.(Based on [18])

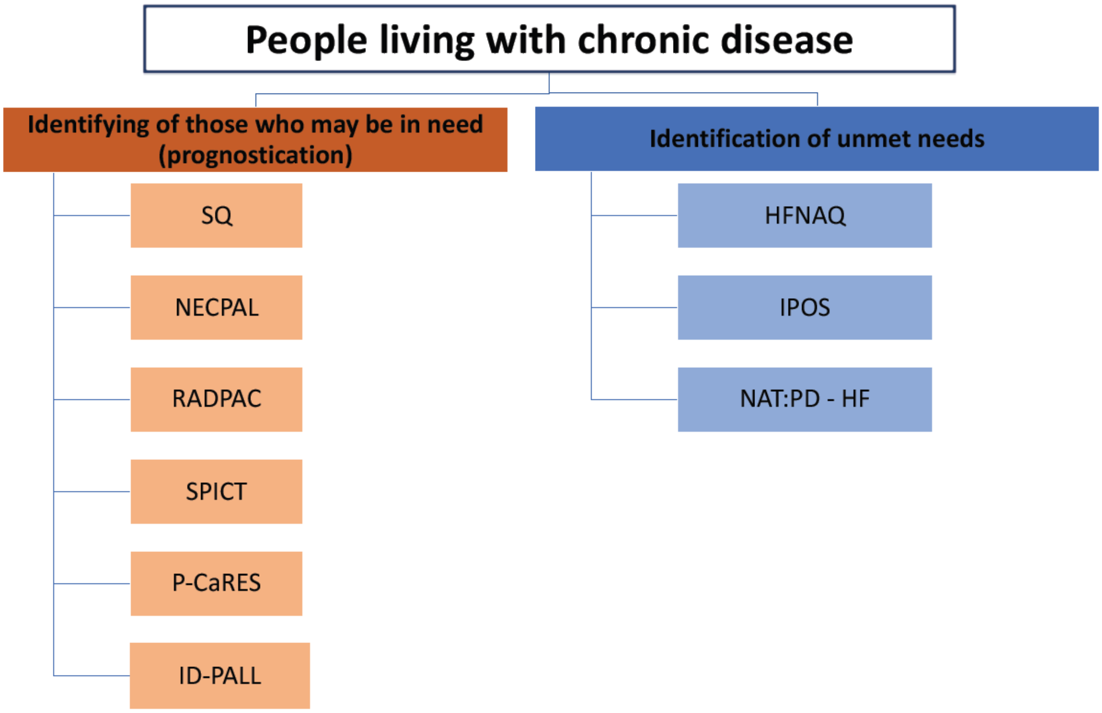

Abbreviations: SQ – **S**urprise **Q**uestion[8]; NECPAL, **Nec**esidades **Pal**liativas Programm[9], RADPAC, **Rad**bound indicators of **P**alliative **C**are needs[10]; SPICT, **S**upportive and **PC** **I**ndicators **T**ool[11], P-CaRES, Palliative Care and Rapid Emergency Screening[12], ID-PALL, **ID**entification of patients in need of **PALL**iative care[13], HFNAQ, Heart Failure Needs Assessment Questionnaire[14], IPOS - Integrated Palliative care Outcome Scale[15], NAT:PD–HF, **N**eeds **A**ssessment **T**ool: **P**rogressive **D**isease – **H**eart **F**ailure[17]

Figure 2. The IDentification of patients in need of PALLiative care (ID Pall).[16]

ID-PALL© G

IDentification of patients in need of **G**eneral **Pall**iative Care

General palliative care is provided by professionals without specialized palliative care training in all care settings and contexts

Please respond to all of the statements below relative to the patient's **current situation**:

1. Would you be surprised if this patient died in the next **12** months?	☐ Yes ☐ No
2. The patient has a progressive illness or group of illnesses or comorbidities that **limits their life expectancy**<br><br>**AND** presents (select all that are applicable):	☐ Yes ☐ No

**a decline in general functioning** (with limited reversibility and an increase in need for support in day to day activities)<br>OR<br><br>a **pronounced instability over the last 6 months** (defined by: one uncontrolled symptom from the patient's point of view OR a pressure ulcer category ≥3 OR more than one acute delirium episode, infection, unscheduled hospitalisation or fall)<br>OR<br>**psychosocial or existential suffering** (of the patient or people close to them)<br>OR<br>**the need for support** in making decisions during the final stages of life	☐ Yes ☐ No<br><br>☐ Yes ☐ No<br><br>☐ Yes ☐ No
3. Current or planned interruption of treatments with curative intent or **vital support measures** (e.g.: artificial ventilation, dialysis, artificial feeding, or hydration)	☐ Yes ☐ No
4. Request for **comfort care or palliative care** from the patient, people close to them, or health professionals	☐ Yes ☐ No

If you have ticked:

NO to question 1 OR YES to at least ONE of the statements 2, 3, or 4, the patient is likely to require general palliative care.

Please complete the ID-PALL S questionnaire on the next page and refer to the general palliative care practice recommendations.

ID-PALL© S

IDentification of patients in need of **S**pecialist **Pall**iative Care.

Specialized palliative care is provided by or with professionals specialized in palliative care.

Please respond to all of the statements below, relative to the patient's **current situation**, **only when the response to the ID-PALL G is positive:**

1. Presence of **at least one severe and persistent symptom**, including pain, that has not responded satisfactorily to treatment within 48 h.	☐ Yes ☐ No
2. **Difficulties in evaluating** physical symptoms or psychological, social difficulties or spiritual distress	☐ Yes ☐ No
3. **Disagreement or uncertainty** on the part of the patient, people close to them or health professionals regarding, for example, medical treatments, resuscitation code or complex decisions	☐ Yes ☐ No
4. The patient has **severe psychosocial or existential suffering** (e.g,: marked symptoms of anxiety or depression, feelings of isolation or of being a burden, loss of meaning or hope, desire to die, or has made a request for assisted suicide)	☐ Yes ☐ No
5. People close to the patient experience **severe psychosocial or existential suffering** (e.g.: marked symptoms of anxiety or depression, major feelings of	☐ Yes ☐ No

exhaustion, major disruption to the functioning of the family system, loss of meaning or hope)	
6. **Palliative sedation** is envisaged (to relieve an intolerable refractory symptom by decreasing the level of consciousness using specific medication)	☐ Yes ☐ No
7. **Advance care plan or advance directives** are difficult to establish with the patient and/or people close to them	☐ Yes ☐ No
8. In your opinion, the patient, people close to them or health professionals could benefit from the **intervention of palliative care specialists**.	☐ Yes ☐ No

If you have ticked **YES to ONE of the above statements,** the patient is likely to benefit from a consultation with a specialist PC team.

Suppl. Figure 3. The Needs Assessment Tool: Progressive Disease – Heart Failure (NAT:PD-HF).[17]

NEEDS ASSESSMENT TOOL : PROGRESSIVE DISEASE HEART FAILURE (NAT: PD-HF)

COMPLETE ALL SECTIONS

PATIENT NAME: ______________________

DATE: __________ DIAGNOSIS: ______________

PATIENT/ADDRESS LABEL

SECTION 1: PRIORITY REFERRAL FOR FURTHER ASSESSMENT

	Yes	No	If yellow boxes are ticked, consider assessment by SPCS
1. Does the patient have a caregiver readily available if required?			
2. Has the patient or caregiver requested a referral to a specialist palliative care service (SPCS)?			
3. Do you require assistance in managing the care of this patient and/or family?			

SECTION 2: PATIENT WELLBEING (Refer to the back page for assistance)

	Level of Concern			Action Taken		
	None	Some/ Potential	Significant	Directly managed	Managed by other care team member	Referral required
1. Is the patient experiencing unresolved physical symptoms (including problems with breathlessness, pain, fatigue, nausea, oedema, insomnia or cough)?						
2. Does the patient have problems with daily living activities?						
3. Does the patient have psychological symptoms that are interfering with wellbeing or relationships?						
4. Does the patient have concerns about how to manage his/her medication and treatment regimes?						
5. Does the patient have concerns about spiritual or existential issues?						
6. Does the patient have financial or legal concerns that are causing distress or require assistance?						
7. From the health delivery point of view, are there health beliefs, cultural or social factors involving the patient or family that are making care more complex?						

8. Does the patient require information about: (tick any options that are relevant)
☐ The prognosis ☐ Treatment options ☐ Heart disease ☐ Advance directive/resuscitation preferences ☐ Medical/health/support services ☐ Financial/legal issues ☐ Social/emotional issues

COMMENTS: ______________________

SECTION 3: ABILITY OF CAREGIVER OR FAMILY TO CARE FOR PATIENT (Refer to the back page for assistance)

Who provided this information? (please tick one) ☐ Patient ☐ Caregiver ☐ Both	Level of Concern			Action Taken		
	None	Some/ Potential	Significant	Directly managed	Managed by other care team member	Referral required
1. Is the caregiver or family distressed about the patient's physical symptoms?						
2. Is the caregiver or family having difficulty providing physical care?						
3. Is the caregiver or family having difficulty coping?						
4. Is the caregiver have difficulty managing the patient's medication and treatment regimes?						
5. Does the caregiver or family have financial or legal concerns that are causing distress or require assistance?						
6. Is the family currently experiencing problems that are interfering with their functioning or inter-personal relationships, or is there a history of such problems?						

7. Does the caregiver require information: (tick any options that are relevant)
☐ The prognosis ☐ Treatment options ☐ Advance directive/resuscitation preferences ☐ What to do in event of patient's death ☐ Medical/health/support services ☐ Social/emotional issues ☐ Heart disease ☐ Financial /legal issues

COMMENTS: ______________________

SECTION 4: CAREGIVER WELLBEING (Refer to the back page for assistance)

Who provided this information? (please tick one) ☐ Patient ☐ Caregiver ☐ Both	Level of Concern			Action Taken		
	None	Some/ Potential	Significant	Directly managed	Managed by other care team member	Referral required
1. Is the caregiver or family experiencing physical, practical, spiritual, existential or psychological problems that are interfering with their wellbeing or functioning?						
2. Is the caregiver or family experiencing grief over the impending or recent death of the patient that is interfering with their wellbeing or functioning?						

COMMENTS: ______________________

IF REFERRAL REQUIRED FOR FURTHER ASSESSMENT OR CARE, PLEASE COMPLETE THIS SECTION

1. Referral to: (Name) ______________________
2. Referral to: (Specialty) ☐ General practitioner ☐ Social worker ☐ Psychologist ☐ Specialist palliative care service ☐ Cardiologist ☐ Other __________
3. Priority of assessment needed: ☐ Urgent (within 24 hours) ☐ Semi-Urgent (2-7 days) ☐ Non-Urgent (next available)
4. Discussed the referral with the client. ☐ Yes ☐ No
5. Client consented to the referral. ☐ Yes ☐ No
6. Referral from: Name: __________ Position: __________ Signature: __________

© Centre for Health Research and Psycho-oncology (2009) NAT:PD-HF. (Except where otherwise noted, this work is licensed under http://creativecommons.org/licenses/by-nc-nd/3.0/)

Suppl. Figure 4. Needs assessment and assignment of responsibility for PC provision (modified from[2])

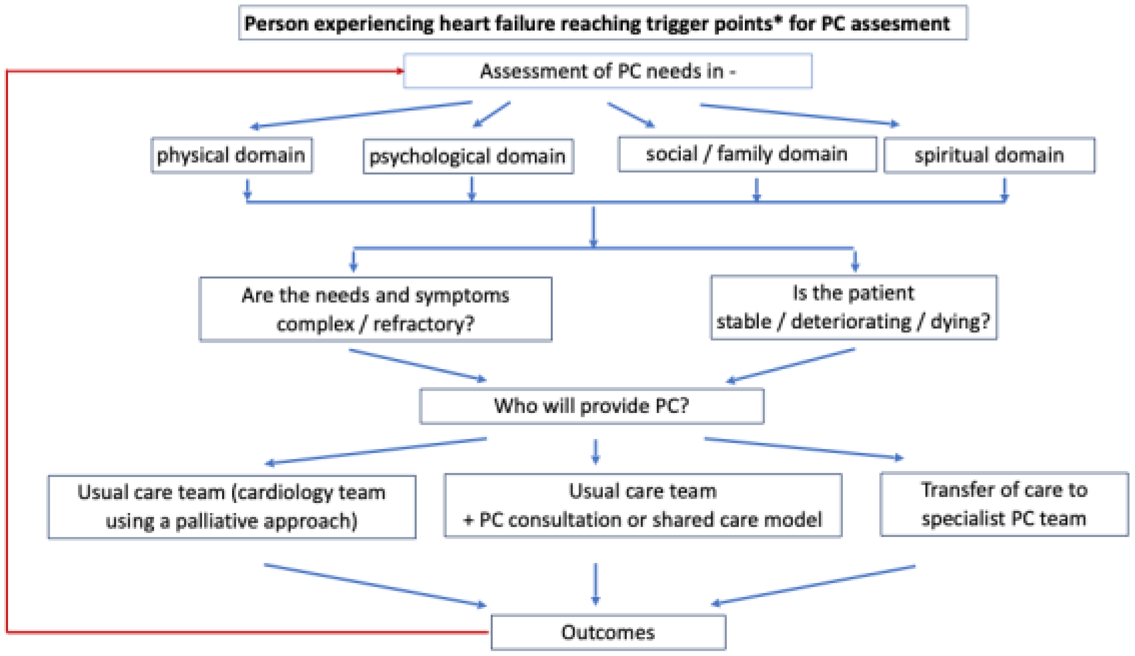

*triggers for performing needs assessment are listed in Table 1.
